# Blood Mitochondrial DNA Content in HIV-Exposed Uninfected Children with Autism Spectrum Disorder

**DOI:** 10.3390/v10020077

**Published:** 2018-02-11

**Authors:** Matthew A. Budd, Kristina Calli, Lindy Samson, Jennifer Bowes, Anthony Y. Y. Hsieh, John C. Forbes, Ari Bitnun, Joel Singer, Fatima Kakkar, Ariane Alimenti, Evelyn J. Maan, M. E. Suzanne Lewis, Carole Gentile, Hélène C.F. Côté, Jason C. Brophy

**Affiliations:** 1 Department of Pathology and Laboratory Medicine, University of British Columbia, Vancouver, BC V6T 2B5, Canada; m.budd@alumni.ubc.ca (M.A.B.); anthony_y_hsieh@hotmail.com (A.Y.Y.H.); 2 Centre for Blood Research, University of British Columbia, Vancouver, BC V6T 1Z3, Canada; 3 Department of Medical Genetics, University of British Columbia, Vancouver, BC V6H 3N1, Canada; kcalli@mail.ubc.ca (K.C.); suzanne.lewis@ubc.ca (M.E.S.L.); 4 Department of Pediatrics, University of Ottawa, Ottawa, ON K1H 8L1, Canada; Samson@cheo.on.ca (L.S.); JBrophy@cheo.on.ca (J.C.B.); 5 Children’s Hospital of Eastern Ontario Research Institute, Ottawa, ON K1H 8L1, Canada; jbowes@cheo.on.ca; 6 Department of Pediatrics, University of British Columbia, Vancouver, BC V6H 3V4, Canada; jcforbes@shaw.ca (J.C.F.); aalimenti@cw.bc.ca (A.A.); 7 Department of Pediatrics, The Hospital for Sick Children, University of Toronto, Toronto, ON M5G 1X8, Canada; ari.bitnun@sickkids.ca; 8 CIHR Canadian HIV Trials Network, Vancouver, BC V6Z 1Y6, Canada; jsinger@hivnet.ubc.ca; 9 School of Population and Public Health, University of British Columbia, Vancouver, BC V6T 1Z3, Canada; 10 Centre Hospitalier Universitaire Sainte-Justine, Université de Montréal, Montréal, QC H3T 1C5, Canada; fatima.kakkar@umontreal.ca; 11 Oak Tree Clinic, BC Women’s Hospital and Health Centre, Vancouver, BC V6H 3N1, Canada; emaan@cw.bc.ca; 12 Women’s Health Research Institute, Vancouver, BC V6H 3N1, Canada; 13 Department of Psychology, Children’s Hospital of Eastern Ontario, Ottawa, ON K1H 8L1, Canada; cgentile@cheo.on.ca

**Keywords:** antiretroviral therapy, genetics, neurodevelopment, prophylaxis, mitochondria, HIV, pediatrics

## Abstract

Long-term outcomes of perinatal exposure to maternal antiretroviral therapy in HIV-exposed uninfected (HEU) children are unknown. However, both HIV antiretroviral therapy and autism spectrum disorder (ASD) have been associated with mitochondrial alterations. Leukocyte mitochondrial DNA (mtDNA) content can serve as a marker for mitochondrial dysfunction. In this cross-sectional, nested case-control study, HEU children with ASD were matched approximately 1:3 on age, sex, and ethnicity to HEU children without ASD, HIV-unexposed uninfected (HUU) controls, and HUU children with ASD. Leukocyte mtDNA content was measured using quantitative PCR. Among 299 HEU in this study, 14 (4.7%) were diagnosed with ASD, which is higher than the general population prevalence estimates. HEU children without ASD and HUU children with ASD had higher mtDNA content than HUU controls. HEU children with ASD had significantly higher mtDNA content than all other study groups. Our results suggest a clear association between elevated leukocyte mtDNA content and both HEU and ASD status. This may implicate mitochondrial dysfunction as a contributor to the high ASD prevalence observed in our cohort.

## 1. Introduction

The use of combination antiretroviral therapy (ART) during pregnancy along with postnatal antiretroviral (ARV) prophylaxis has effectively reduced vertical transmission of human immunodeficiency virus (HIV) [1,2]. Accordingly, the number of HIV-exposed, uninfected (HEU) children born worldwide has increased significantly and will continue to rise. Although its preventative benefits are evident, concerns have been raised regarding potential long-term effects of perinatal ARV exposure in HEU infants [2,3,4]. A recent study noted no adverse neurodevelopmental outcomes in HEUs before 12 months of age, but stressed a need for assessment in older cohorts [5]. Nucleoside reverse transcriptase inhibitors have been associated with mitochondrial toxicity in adults [6] and children [7,8,9], and several can cross the placental barrier [10] and/or the blood brain barrier [11], prompting concern that in utero exposure to maternal ARVs could be toxic to mitochondria in the developing fetus, and may be associated with mitochondrial dysfunction (i.e., loss of mitochondrial function in some capacity) [12]. In addition to potentially increased risk of developing various metabolic abnormalities and immune irregularities [13], mitochondrial dysfunction in young children has been linked to autism spectrum disorder (ASD) [14,15,16]. There is also abundant evidence of an increased risk of adverse neurodevelopmental problems associated with maternal immune activation and infection during pregnancy [17,18,19,20].

Elevated levels of mitochondrial DNA (mtDNA) per cell in both brain tissue and blood have been associated with advanced age and/or markers of cellular senescence, suggestive of a physiological response to oxidative stress [21,22]. Excess endogenous buildup of reactive oxygen species may induce mitochondrial dysfunction directly through a number of mechanisms, such as damage to mitochondrial proteins, enzymes, lipids, mtDNA, and nuclear DNA (nDNA) [23].

As of 2010, the prevalence of ASD in U.S. children (aged eight years) was estimated at 1.47% [24]. However, in a study of 158 HEU children followed at one Canadian pediatric HIV clinic [25], a higher prevalence of ASD (9/158 or 5.7%, 95% confidence interval (CI): 2.6% to 10.8%) was recently observed. Many of these children were also enrolled in the prospective, pan-Canadian Children & Women AntiRetrovirals & Markers of Aging (CARMA) cohort study. Our aim was to identify children with ASD in this cohort of HEUs, and to compare blood mitochondrial DNA mtDNA content (the ratio of mtDNA to nDNA), a marker for mitochondrial dysfunction, between HEU and HIV-unexposed uninfected (HUU) children with and without a diagnosis of ASD. As we previously observed higher blood mtDNA in HEU neonates compared to HUUs [21], we hypothesized that HEU would have a higher level of mtDNA than HUU, and that within the HEU and HUU groups, children with ASD would have higher levels of mtDNA than corresponding non-ASD controls. Potential relationships between HEUs’ exposure to ARVs (type and duration) and mtDNA content were also explored.

## 2. Materials and Methods

### 2.1. Study Design

A nested case-control study design was used, whereby HEU children with a diagnosis of ASD (detailed in the next section) were matched 1:3 to each of the following groups: (a) HEU children without ASD, matched on sex, ethnicity, and age; (b) HUU anonymous controls and HUU siblings of HUU children with ASD, each matched on sex and age; (c) HUU children with ASD matched on sex, age, and whenever possible, ethnicity.

### 2.2. Study Participants

Paediatric HEU participants in this study were participants of the CARMA cohort study, which prospectively enrolls both children living with HIV and HEU children at four sites across Canada and investigates the effects of HIV and ARVs on markers of cellular aging and HIV comorbidities in women and children [26].

HUU children with ASD were participants in the British Columbia Autism Spectrum Interdisciplinary Research (ASPIRE) Program of ASD-CARC (the ASD-Canadian-American Research Consortium, http://www.autismresearch.ca/). HUU anonymous controls were children who had routine blood work during a visit to the emergency department of the British Columbia Children’s Hospital. For these controls, only sex and age were known and they were assumed to not have ASD. Finally, a small number of non-ASD HUU were siblings of ASPIRE participants, included to allow for adequate numbers of matched case-controls.

Clinical and demographic information was retrieved from the CARMA and ASPIRE databases. Demographic and anthropometric data collected by CARMA included the child’s date and country of birth, estimated gestational age at birth, sex, ethnicity (as reported by self or parents), and paternal and maternal dates of birth. For these analyses, children of mixed background were categorized according to their non-Caucasian ancestry. Clinical data collected included platelet count, type and duration of any perinatal ART exposure, and any maternal substance use the child was exposed to in pregnancy (e.g., tobacco, alcohol, illicit drugs) if available. In addition, health issues in the child related to such factors as gastrointestinal health, psychological conditions, haematology, or other conditions requiring hospitalization were recorded. Data captured by ASPIRE includes in-depth birth and medical histories, original ASD diagnostic assessments, and family histories followed by comprehensive clinical genetics assessment in order to rule out known mitochondrial or nuclear genomic disorders.

Approval for the study was obtained from the Research Ethics Boards of all participating institutions (University of British Columbia research ethics board #H08-02018; approved 24 October 2008).

### 2.3. Diagnosis and Severity of Autism Spectrum Disorder

For the purposes of this study, determination of ASD required a formal diagnosis by an accredited health provider, using the criteria of the fourth or fifth edition of the American Psychiatric Association’s Diagnostic and Statistical Manual of Mental Disorders (typically including assessment with the Autism Diagnostic Observation Schedule and the Autism Diagnostic Interview). All children were screened and diagnosed with ASD as part of routine clinical care and practice (i.e., routine yearly surveillance of children for general health and neurodevelopment post-HIV/ARV exposure), which included general pediatric screening for neurodevelopmental milestones. Diagnoses of ASD for children in both CARMA and ASPIRE were made between 2004 and 2015, between the ages of three and ten years. Formal diagnostic ASD assessments (clinical assessments by developmental pediatricians and/or psychologists’ psychometric assessments) and quantifiable information on ASD severity (such as scores from the Childhood Autism Rating Scale [CARS]) were reviewed when available. However, these scores were not available for all study participants, in particular when these assessments were not available from the hospital health record. Ascertainment of intellectual disabilities and/or developmental delays in HEUs with ASD were based either on standardized psychometric tests (Stanford-Binet, Bayley, Mullen, Vineland, Adaptive Behaviour Assessment Scale), or on clinical assessments by developmental paediatricians and/or psychologists for children who were not testable. For the purposes of analysis, intellectual disability also encompassed assessments of global developmental delay and children with cognitive test scores in the first or second percentile, as well as cases given a formal diagnosis of intellectual disability. To address the severity of ASD, a qualitative descriptor (mild, moderate, severe) was assigned to each child whenever possible. This descriptor was primarily based on CARS scores when available, complemented with clinical assessments by experienced pediatricians and/or mental health experts, informed by all test results available in the health records.

### 2.4. Specimen Collection and Preparation

CARMA HEU whole blood (WB) was collected between January 2010 and April 2015; ASPIRE WB was collected between 2002 and 2014; anonymous HUU WB was collected between April 2010 and December 2011. For HEUs with ASD, WB specimens drawn closest to the date of ASD diagnosis were used; if the date of ASD diagnosis was unknown, the most recent blood specimen was used. Collection, processing, and DNA extraction from CARMA WB specimens have been previously described [26,27]; anonymous controls’ DNA were extracted in the same manner as the CARMA WB. Extracts of WB DNA from HUU ASD children and non-ASD siblings were obtained from the ASPIRE biobank. All blood specimens were processed within 48 h of collection; we have previously ascertained that blood mtDNA content is stable over this period of time.

### 2.5. MtDNA Content Assay

WB DNA extracts were diluted 1:10 with elution buffer AE (Qiagen, Hilden, Germany) at pH 9.0, containing 10 mM Tris-Cl and 0.5 mM ethylenediaminetetraacetic acid (EDTA). MtDNA content was measured using a monochrome, multiplex quantitative polymerase chain reaction (qPCR) assay similar to a method published for relative telomere length determination, allowing for the quantification of two genes in the same well [28]. MtDNA content was expressed as the ratio between mtDNA copy number (D-loop region) and the copy number of a single-copy nuclear gene (albumin), previously shown by members of our group to be suitable for use in a multiplex assay [27]. The primer sequences used are shown in Supplementary Materials Table S1.

DNA extracts were assayed on the LightCycler^®^ 480 platform (Roche, Basel, Switzerland). For each reaction, 2 μL of DNA was added to 8 μL of master mix for final concentrations of 1× FastStart SYBR Green Master (Roche), 1.2 mM EDTA, and 4 primers at 0.9 μM (Supplementary Materials Table S1). All extracts were assayed in duplicate, and each plate included a standard curve, a negative control, and two internal controls. The standard curve was generated by 1:5 serial dilutions of two cloned plasmids containing either the albumin or D-loop amplicon, and mixed in a 1:50 ratio. Matched children were assayed on the same plate to reduce inter-assay bias when performing between-group comparisons, and well positions were randomized. All DNA extracts used were kept blind to the research team until final analysis. The thermal cycling profile is shown in Supplementary Materials Table S2, and was similar to that previously used for telomere length determination [28].

### 2.6. Statistical Analyses

Two-tailed Student’s *t*-tests or Mann-Whitney U-tests were used to compare mtDNA content between groups, after ascertaining the normality of data within each group via the Shapiro-Wilk test. One group’s data (HEU with ASD) was not normally distributed even after log-transformation. To account for performing multiple two-group comparisons, we re-performed analyses of mtDNA content using the Kruskal-Wallis one-way analysis of variance, including data from all four study groups. This was followed by post-hoc testing using Dunn’s method to correct for multiple comparisons. Confidence intervals for ASD prevalence were estimated using a Poisson distribution. All analyses were conducted using the R computing environment, v3.4.3 (R Foundation for Statistical Computing, Vienna, Austria). A *p*-value <0.05 was considered statistically significant.

### 2.7. Sensitivity Analyses

Due to an ethnicity mismatch between CARMA and ASPIRE children, and to account for the unknown ethnicities of many HUU controls, a sensitivity analysis was performed to examine whether mtDNA content differed significantly between sex and age-matched Asian/South Asian, Caucasian, and African-Canadian ethnic groups. CARMA HEU children without ASD of Asian or South Asian ethnicity were matched 1:2 with non-ASD HEU children of Caucasian (*n* = 20) and African-Canadian (*n* = 20) ethnicity. None of the children included in this analysis were included in the main study. All samples were blinded and randomized in the same manner as in the main arm of the study, with matched samples assayed on the same plate. Between-group comparisons of mtDNA content were done using two-tailed Student’s *t*-test. We performed a sample size calculation to estimate the number of participants needed in each group to detect a difference with 80% power.

We also performed a sensitivity analysis for potential bias due to differential dilution factors of our DNA extracts. For those extracts with both an albumin copy number and mtDNA content value within acceptable quality control ranges (*n* = 147), we examined the correlation between albumin copy number and mtDNA content with Spearman’s rank correlation coefficient and modeled the relationship with simple linear regression. We then diluted a separate aliquot of each DNA extract to achieve a common concentration of albumin across all extracts and repeated the assay. We used Pearson’s product moment correlation and linear regression to determine whether our results were affected by dilution factor by comparing mtDNA content of our extracts before and after this dilution.

## 3. Results

Among HEU children enrolled in the CARMA cohort as of December 2015, 14/299 (4.7%, 95% CI: 2.6% to 7.7%) had a confirmed diagnosis of ASD. Of note, ASD prevalence among CARMA children living with HIV was 2/144 (1.4%, 95% CI: 0.2% to 5.0%).

Basic demographic characteristics of HEU children with and without ASD, and HUU children with and without ASD are shown in Table 1. Among all HEUs, there were three pairs of siblings and one pair of half-siblings (same mother) included in the study; of these, one sibling pair and one of the half-siblings received a diagnosis of ASD. Maternal age at birth was available for 41/42 (98%) of HEUs without ASD, 9/51 (18%) of HUUs without ASD, and all children in other groups. Paternal age at birth was available for 11/14 (79%) of HEUs with ASD, 38/42 (90%) of HEUs without ASD, 41/42 (98%) of HUUs with ASD, and 9/51 (18%) of HUUs without ASD. Maternal and paternal age did not differ significantly between groups.

Maternal ART information was available for 54/56 (96%) of HEU children. Of those children with maternal ART information available, 49/54 (90%) were exposed to ARVs in utero. A summary of maternal regimens is shown in Table 2. The majority of mothers who received ART during pregnancy were treated with a combination regimen of two nucleoside reverse transcriptase inhibitors and either (a) a protease inhibitor (37/49, 76%), or (b) a non-nucleoside reverse transcriptase inhibitor (3/49, 6%). If the mother switched regimens during pregnancy, the regimen taken for the majority of pregnancy was reported.

**Figure 1:**
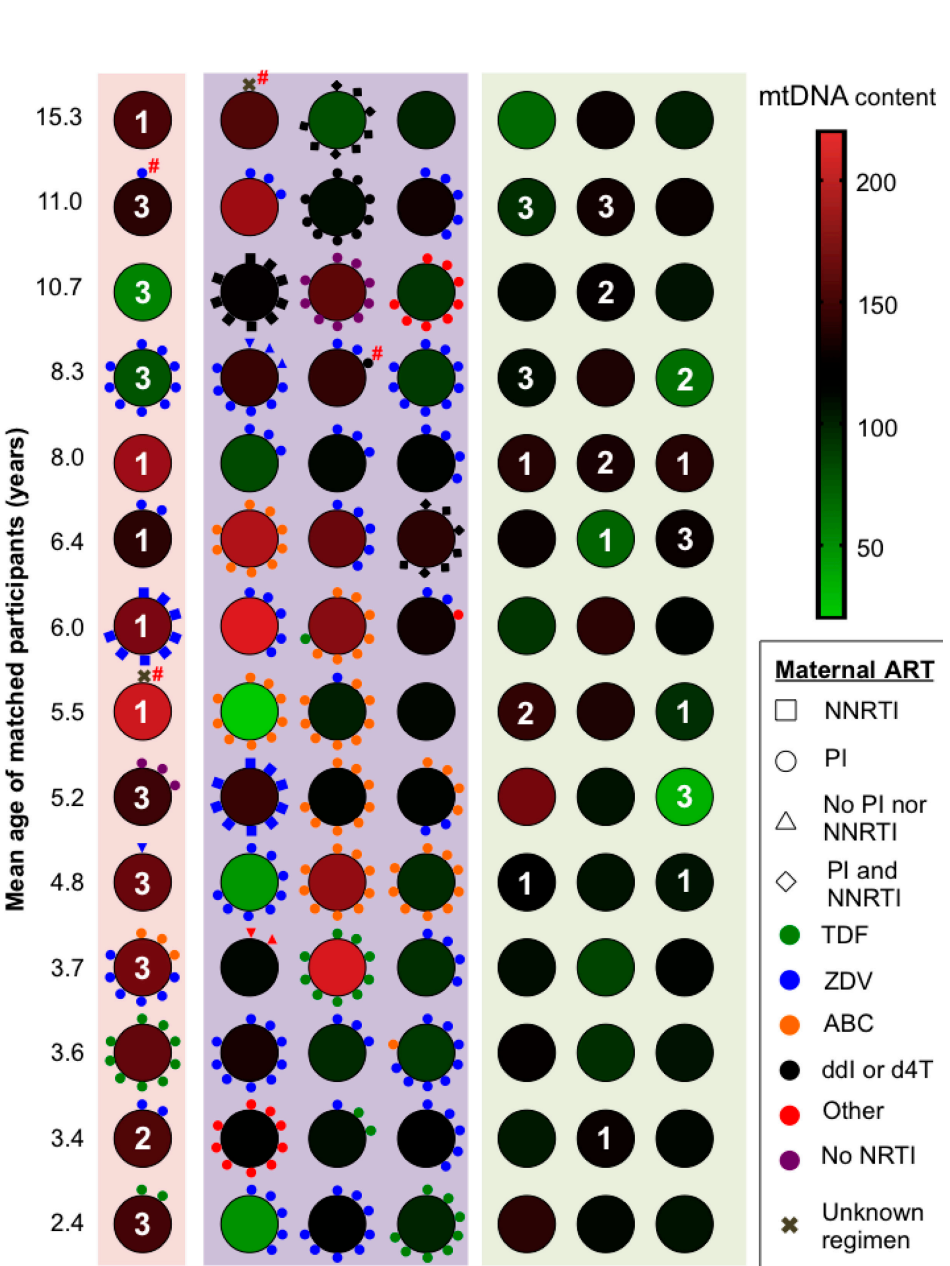
Graphical representation of mitochondrial DNA (mtDNA) content, maternal antiretroviral therapy (ART) parameters, and severity of autism spectrum disorder (ASD) in 98 study participants. White numbers reflect ASD severity: 1 = mild or mild/moderate; 2 = moderate; 3 = moderate/severe or severe. Satellite shapes (square, circle, triangle, diamond) indicate non-nucleoside reverse transcriptase inhibitor (NNRTI) drug exposure, and color indicates exposure to NRTI backbone (one per month, clockwise arrangement).

The most commonly used individual ARVs were lamivudine (44/54, 81%), zidovudine (33/54, 61%), nelfinavir (21/54, 39%), ritonavir-boosted lopinavir (17/54, 31%), and abacavir (14/54, 26%). Notably, 3/14 (21%) HEUs with ASD received no exposure to ARVs in utero, compared to 2/42 (5%) HEUs without ASD (Fisher’s exact *p* = 0.094). However, HEU children with ASD had a shorter duration of exposure to maternal ART than HEUs without ASD (11 vs. 33 weeks, Table 2) and consequently may have experienced greater exposure to maternal HIV “milieu”. A visual representation relating mtDNA content, maternal ART parameters, and ASD severity is shown in Figure 1.

### 3.1. MtDNA Content Analyses

**Figure 2:**
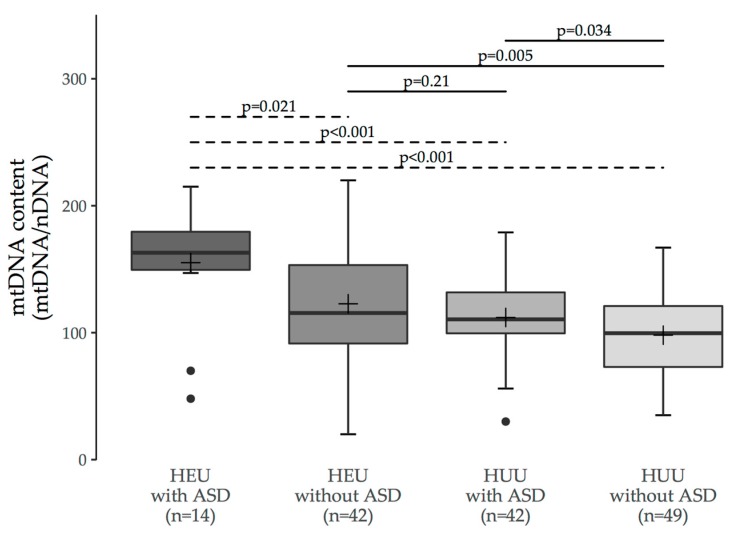
Between-group comparisons of blood mtDNA content in HIV-exposed uninfected (HEU) and HIV-unexposed uninfected (HUU) children with and without ASD. *p*-Values are reported for Student’s *t*-test (solid lines) or Mann-Whitney U-test (dashed lines).

Blood mtDNA content (mtDNA/nDNA ratio) was obtained for 149 study participants (Figure 2). For two DNA extracts (both HUU controls), the measures were outside the known range of linearity of the assay over two independent runs; these were therefore excluded, as per a priori quality control criteria. To evaluate the association between ASD and mtDNA content, we compared measurements between ASD and non-ASD children within each of the HEU and HUU groups. HEU children with ASD had higher mtDNA content (median [interquartile range (IQR)]: 163 [150 to 180], *n* = 14) than HEU children without ASD (116 [92 to 153], *n* = 42; *p* = 0.021). Similarly, HUU children with ASD had higher mtDNA content (110 [100 to 132], *n* = 42) than HUU children without ASD (100 [73 to 121], *n* = 49; *p* = 0.034).

To evaluate the association between HIV/ARV exposure status and mtDNA content, we compared measurements between HEU and HUU children in each of the ASD and non-ASD groups. HEU children with ASD had higher mtDNA content than HUU children without ASD (*p* < 0.001), and HEU children without ASD had higher mtDNA content than HUU children without ASD (*p* = 0.005).

MtDNA content of the HUU anonymous controls (*n* = 40) and non-ASD HUU siblings of ASD children (*n* = 9) were similar (*p* = 0.70, Student’s *t*-test; difference between means [95% CI] = 5 [−19 to 29]). Overall results were unchanged if HUU non-ASD siblings were omitted from the analysis. For those HEU participants for whom platelet counts were available in the CARMA database (*n* = 54/56), there was no correlation between platelet count and mtDNA content (Pearson’s *r* = 0.08, *p* = 0.55). A summary of all between-group comparisons of mtDNA content is shown in Figure 2.

### 3.2. Multiple Group Comparisons

The Kruskal-Wallis omnibus *p*-value was <0.001; *p*-values for pairwise comparisons calculated via Dunn’s post-hoc test are shown in Table 3. Note that the Bonferroni correction was not applied to the significance level for this analysis in order to reduce the risk of type II error in this exploratory study. *p*-values for all two-group comparisons were comparable to those from the Mann Whitney U-test (Table 3; Figure 2).

### 3.3. Sensitivity Analyses

#### 3.3.1. Ethnicity

A summary of the groups used in this sensitivity analysis is shown in Table 4. Overall, the children included in this sensitivity analysis were younger than those in the main study due to the constraints of our matching coupled with the relatively small remainder of HEU participants who were not in the main arm.

**Figure 3:**
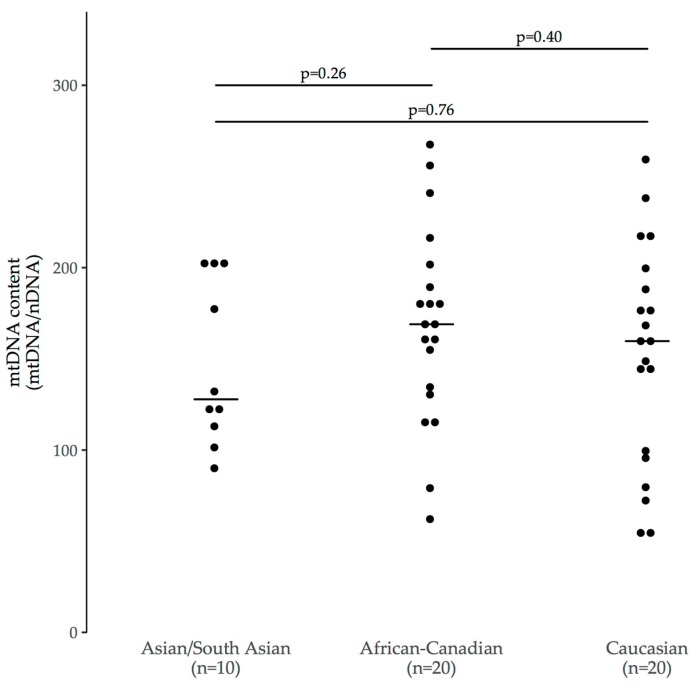
Univariate between-group comparisons for three sex- and age-matched (1:2) groups of HEU children without ASD (*p*-values calculated via Student’s *t*-test). Lines indicate medians.

There were no significant mtDNA content differences observed between any of the three groups (*p* = 0.26 to 0.76, Figure 3). We used Cohen’s d as an estimate of effect size between groups (power = 0.80, α = 0.05). We found that between Caucasian and Asian/South Asian children (*d* = 0.12), a sample size of *n* = 1177 in each group would provide 80% power to detect a difference in mtDNA content. Similarly, between African-Canadian and Caucasian children (*d* = 0.27), group sizes of *n* = 216 each would be required, and between Asian/South Asian and African-Canadian children (*d* = 0.44), group sizes of *n* = 83 each would be required. Notably, the African-Canadian and Caucasian groups had median values similar to those of HEU children with ASD (median [IQR]: 163 [150 to 180]).

#### 3.3.2. DNA Extract Dilution Factor

**Figure 4:**
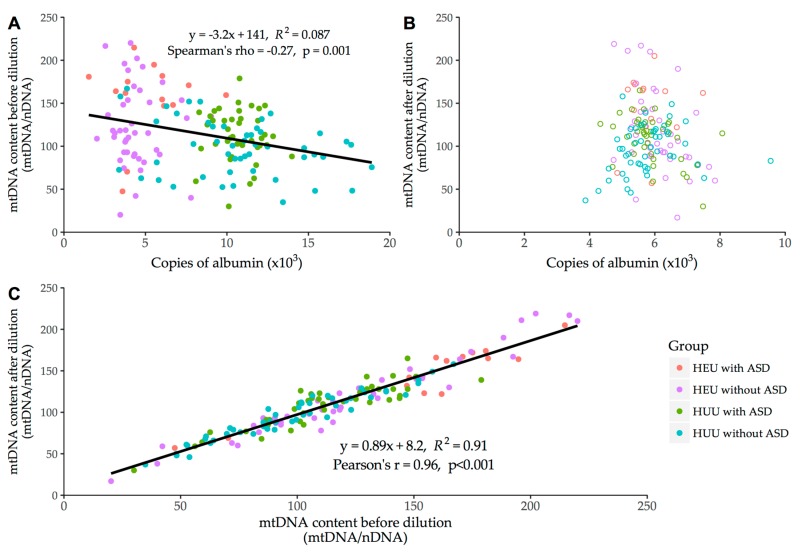
Correlation of mtDNA content and albumin copy number before (panel **A**) and after dilution to a common albumin concentration (panel **B**). Linear correlation of mtDNA content measurements before and after systematic dilution (panel **C**).

The results of this analysis are presented in Figure 4. Extracts were diluted to approximately 5000 to 7000 copies of albumin per 2 μL (mean (standard deviation) [range] = 5906 (806) [3872–9551]). The correlation coefficient, regression equation, and R^2^ of this model suggest that results were reproducible irrespective of dilution factor and were not affected by systematic shift (Figure 4C).

### 3.4. Clinical Outcomes

Our study was not designed nor powered to investigate clinical outcomes. Although not statistically significant, the data may suggest a pattern whereby the severity of ASD symptoms appeared to be greater in HEUs than HUUs. Among children for whom psychometric test scores and formal assessments of development were available (*n* = 56 HEU, *n* = 47 HUU), 9/12 (75%) of HEU with ASD and 22/38 (58%) of HUUs with ASD were identified as intellectually disabled or showing signs of global developmental delay. Assessments of mild, moderate, or severe ASD as categorized by the Childhood Autism Rating Scale were available for 9/14 HEUs with ASD and 8/42 HUUs with ASD. Delays in language development not otherwise accompanied by cognitive deficits were also noted (Table 2). In total, 11/12 (92%) of HEUs with ASD and 28/38 (74%) of HUUs with ASD showed signs of substantial language and/or cognitive delay. Rates of intellectual disabilities in both HEU and HUU children with ASD were similar to those of the entire ASPIRE cohort (200/337, 59%). Among the HEU children with ASD for whom cognitive and developmental test scores (*n* = 10) from clinical diagnostic assessments by developmental pediatricians and/or psychologists were available, nine had scores in the first or second percentile for cognitive skills (Bayley Scale of Infant Development, Mullen Scale of Early Learning, or Stanford-Binet Intelligence Scale) and/or adaptive intelligence (Vineland Adaptive Behaviour Scale, Adaptive Behaviour Assessment System), suggesting that these children met criteria for intellectual disability. Only one child was noted as being of normal cognitive ability, but was assessed as having specific areas of weakness in verbal skills and short-term memory.

Among HUUs with ASD, 26/42 (62%) were unable to be assessed or did not have a specified severity. Common reasons for these children to not be assessed were that they were not yet developmentally advanced enough for testing or evaluation (7/13, 54%), had a mixed cognitive profile (3/13, 23%), or were uncooperative and/or inattentive during testing (3/13, 23%). HUU children who did not have a severity rating specified generally did not meet criteria for intellectual disability and thus did not undergo evaluation (11/13, 85%), although 2/13 (15%) cases were noted by experts as being suspected of functioning in the intellectual disability range but had not been evaluated.

## 4. Discussion

In this study, HIV/ARV exposure, irrespective of ASD diagnosis, was associated with elevated blood mtDNA content compared to HUU controls. We have ascertained that this finding is likely not confounded by platelet count, as has been previously described in certain studies [29,30,31], nor by differences in the concentrations of the DNA extracts themselves (Figure 4). This observation is consistent with previous studies, by both our group [21] and others [32,33], but is in contrast with some reports of decreased mtDNA content in HEU children [34,35]. However, there is heterogeneity among these studies with regard to the ARVs to which children were exposed, the timing of mtDNA measures reported, and whether measurements were made before, during, or after infant prophylaxis. To explain the elevated mtDNA content in HEUs, some have suggested that there may be a physiological rebound effect in mtDNA content after removal of the pressure exerted by HIV and/or ARV exposure. For example, one study reported low mtDNA content in HEU children early in life, that by two to five years of age had rebounded to values close to those of controls [34]. This effect was also observed by our group, whereby HIV patients treated with certain ARVs experienced mtDNA depletion that was reversed upon treatment discontinuation or change to less toxic regimens [6,36]. Our group has previously reported increases in mtDNA content in ARV-exposed HEU children at birth (consistent with findings from other groups [32]), which persisted months after the ARV prophylactic period (first six weeks of life), and is also consistent with our current findings [21]. This would also partially explain why two of the three groups in our sensitivity analysis for ethnicity showed mtDNA content values close to those for HEU children with ASD, as the children in the sensitivity analysis were generally much younger (approximately 1–2 years of age (Table 4) in the sensitivity analysis versus approximately 6 years of age in the main study (Table 1)). Another possible explanation for this finding is that mtDNA haplogroup variation and genetic ancestry may play a contributory role, along with environmental factors, in the development of ASD according to recent findings [37]. It has been shown that self-identified ethnicity may not fully encapsulate the complex nature of haplogroup heterogeneity and it may not be a good predictor of one’s genetic make-up [38,39,40,41], pointing to a more nuanced relationship between mtDNA, ASD progression, and ethnicity than the results presented in this exploratory analysis.

ASD diagnosis in both the HEU and HUU groups was also associated with elevated blood mtDNA content, a finding that is consistent with previous literature [14,42]. It may be that a genetic predisposition for ASD, mediated by mitochondrial dysfunction, is exacerbated by HIV/ARV exposure leading to a higher level of mitochondrial dysfunction and thus greater penetrance of ASD symptoms. These findings may also indicate that ASD, which has been associated with signs of chronic oxidative stress [43] and mitochondrial dysfunction [15,44], is associated with a persistent deleterious effect on mitochondria that may induce mitochondria biogenesis as a compensatory mechanism. This would be consistent with the apparent cumulative effect we report in HEU with ASD.

Although group sizes for individual ART regimens were small (see Table 2) and precluded quantitative analysis, it is noteworthy that HEU children with ASD tended to have shorter exposure to maternal ART than HEU children without ASD. This could implicate prolonged perinatal HIV “milieu” exposure as a possible predictor for ASD among HEU children. However, this would not be consistent with the finding that only 2/144 (1.4%) of CARMA’s HIV-infected children were diagnosed with ASD. Most of these children were born to mothers with either untreated or poorly controlled HIV, therefore exposed to elevated viral load. It may be that certain maternal comorbidities and/or substance exposures led to a compounded risk of ASD in their child when combined with ARV or HIV exposure in pregnancy. Alternatively, this could suggest that initiating ART later in pregnancy may increase the risk of ASD. At least one previous study suggested increased risk of mitochondrial dysfunction in HEU when ART was started late in pregnancy [45]. More work is needed to elucidate any potential specific associations with different maternal ARV combinations, initiation times, and adherence parameters.

### Limitations

Our study is subject to the limitations of observational studies. An important caveat of this study is that we cannot compare the prevalence of ASD within our cohort with that reported in the general population because of possible bias during recruitment of participants to the CARMA cohort. More specifically, there was a possible bias toward enrolling HEU children with ASD from 2013 to present, once it was reported that ASD prevalence among HEUs appeared elevated [25]. At face value, this could artificially inflate the prevalence of ASD in HEU children reported here. However, there was also evidence of a clear bias against recruiting HEU children with ASD before this time, as the children typically become distressed during blood draws, such that families of ASD children were less likely to be invited to participate and/or deemed more likely to decline participation. It should be noted that half (7/14) of the HEU children with ASD in this study were recruited during each period. Overall, we ascertain that enrollment bias in recent years was likely counter-balanced by a reverse bias during the early years of the CARMA study, but this remains a limitation that prevents us from reaching conclusions with respect to the prevalence result. Another factor limiting our interpretation of this prevalence observation is the convenience sampling of participants who may be undergoing closer clinical observation than the general population due to the risks of perinatal HIV exposure. We acknowledge that identification, screening, and presentation of ASD among these patients is likely to be variable and future studies should apply a more standardized methodology for case selection. Nevertheless, it provides a strong impetus for other studies to investigate the population-based prevalence of ASD in the HEU population, as well as to evaluate ASD severity in this group.

One of the key limitations of our study is the lack of consistently available information relevant to the diagnosis, evaluation, and monitoring of variables relevant to ASD. Many HUU children with ASD did not receive an assessment of severity for ASD or intellectual disability, or were not assessed due to a lack of intellectual impairment. Among HEUs, we were limited to the information available to us in clinical charts, which commonly did not include information regarding psychometric and neurodevelopmental assessment. This prevents us from commenting on or comparing severity between groups; this is something that should be evaluated in future studies involving HEU children.

The assumption that children in the anonymous HUU control group do not have ASD cannot be assured as only sex and age data were available. However, given our observations that ASD and HEU children had elevated mtDNA content compared to controls, any ASD or HEU cases among this anonymous group would likely present a conservative bias. Ethnicities were unevenly distributed amongst groups, and ethnicity data was unavailable for much of the non-ASD HUU group. However, the sensitivity analysis we performed suggests that potential differences in ethnicity were likely not meaningful confounders of our results.

While we demonstrated an association between HEU status and elevated mtDNA content, it is unclear whether this is modulated by the effects of HIV or ARV exposure. Information about the mothers, including prior to or during pregnancy, was often limited when children were enrolled after infancy. Thus, we lack detailed measures of maternal comorbidities, non-HIV medications and substance use in pregnancy, as well as detailed family mental health histories. This lack of information prevents us from commenting on between-group maternal differences that may exist on such factors as maternal psychotropic mediation use, psychiatric conditions, or smoking during pregnancy, and may confound our results. Similarly, information about maternal HIV viral load or CD4 count during the perinatal period was often unavailable. We were also not powered to explore differential associations with individual ARV agents.

The prevalence of ASD among HEU children in the CARMA cohort is approximately three times that seen in the general population. If this finding were to be replicated in other cohorts, it would necessitate the conduct of large prospective cohort studies to determine the etiology of the association.

## 5. Conclusions

HIV exposure and ASD diagnosis were both independently associated with elevated blood mtDNA content in children and adolescents between the ages of two and 16 years. In children with both HIV exposure and ASD, mtDNA content was further elevated. These results clearly warrant further investigation into the possible effects of maternal, clinical, demographic, environmental, and other factors, and to validate whether these findings are reproduced in other cohorts. Furthermore, the prevalence of ASD within our HEU cohort is concerning and stresses the need for longer follow-up and further research on the long-term neurodevelopmental outcomes in the rapidly growing HEU population worldwide [46].

## Figures and Tables

**Table 1:** Demographic characteristics of the matched study participants (cases and controls).

Characteristic	HEU with ASD *n* = 14	HEU without ASD *n* = 42	HUU with ASD *n* = 42	HUU without ASD *n* = 51
Male sex	10/14 (71)	30/42 (71)	30/42 (71)	36/51 (71)
Age at blood collection (years)	6 (4–8) [2–16]	6 (4–8) [2–16]	6 (4–8) [2–16]	6 (4–9) [2–16]
Ethnicity				
Black/African-Canadian	11/14 (79)	33/42 (79)	6/42 (14)	0/51
White/Caucasian	3/14 (21)	9/42 (21)	22/42 (52)	5/51 (10)
Asian/South Asian	0/14 (0)	0/42 (0)	10/42 (24)	4/51 (8)
Indigenous	0/14 (0)	0/42 (0)	4/42 (10)	0/51 (0)
Unknown (anonymous)	0/14 (0)	0/42 (0)	0/42 (0)	42/51 (82)
Maternal age at birth (years)	30 (24–32) [18–42]	33 (31–36) [25–42]	31 (28–35) [18–42]	27 (26–36) [21–39]
Paternal age at birth (years)	35 (29–42) [25–44]	37 (34–41) [24–63]	35 (30–38) [20–51]	30 (28–38) [26–44]

Data are reported as median (interquartile range) [range] or *n*/*N* (%); HEU—HIV-exposed uninfected; HUU—HIV-unexposed uninfected; ASD—autism spectrum disorder.

**Table 2:** Clinical characteristics of matched study participants (cases and controls).

Characteristic	HEU with ASD *n* = 14	HEU without ASD *n* = 42	HUU with ASD *n* = 42	HUU without ASD *n* = 51
Developmental disorders/delays				
Intellectual disabilities	9/14 (64)	0/42 (0)	22/42 (52)	0/9 (0)
Language delay	2/14 (14)	2/42 (5)	6/42 (14)	0/9 (0)
Unknown or unable to assess	2/14 (14)	0/42 (0)	4/42 (10)	42/51 (82)
Severity of ASD symptoms				
Mild or mild/moderate	5/14 (36)	-	7/42 (17)	-
Moderate	1/14 (7)	-	4/42 (10)	-
Moderate/severe or severe	7/14 (50)	-	5/42 (12)	-
Unable to assess	0/14 (0)	-	13/42 (31)	-
Not specified	1/14 (7)	-	13/42 (31)	-
History of seizures/epilepsy (ever)	2/14 (14)	0/42 (0)	5/42 (12)	0/9 (0)
Low muscle tone	3/14 (21)	2/42 (5)	14/42 (33)	1/9 (11)
Chronic gastrointestinal disorders	10/14 (71)	9/42 (21)	7/42 (17)	0/9 (0)
Maternal ARV regimen during pregnancy ^1^				
Dual NRTIs+PI				
ZDV + 3TC + PI	5/14 (36)	19/42 (45)	-	-
ABC + 3TC + PI	0/14	8/42 (19)	-	-
TDF + FTC + PI	2/14 (14)	3/42 (7)	-	-
Other combinations + PI	1/14 (7)	4/42 (10)		
Dual NRTIs + NNRTI	1/14 (7)	2/42 (5)	-	-
NRTI + NNRTI + PI	0/14 (0)	2/42 (5)		
Other	1/14 (7)	1/42 (2)	-	-
None	3/14 (21)	2/42 (5)	-	-
Unknown	1/14 (7)	1/42 (2)	-	-
Infant received ZDV prophylaxis	13/14 (93)	42/42 (100)	-	-
Length of in utero ARV exposure (weeks) ^2^	11 (6–35) [0–38]	33 (20–39) [0–41]	-	-
Length of neonatal ZDV prophylaxis (weeks)	6 (4–6) [0–7]	6 (6–6) [4–7]	-	-

Data are reported as median (interquartile range) [range] or n/N (%); ARV—antiretroviral; PI—protease inhibitor; (N)NRTI—(non-)nucleoside reverse transcriptase inhibitor; ZDV—zidovudine; 3TC—lamivudine; TDF—tenofovir disoproxil fumarate; FTC—emtricitabine; ABC—abacavir; HEU—HIV-exposed uninfected; HUU—HIV-unexposed uninfected; ASD—autism spectrum disorder; ^1^ Also see Figure 1; ^2^ Maternal ARV exposure time during pregnancy was available for 12/14 HEUs with ASD and 40/42 HEUs without ASD; -: Not applicable.

**Table 3:** *p*-Values for pairwise comparisons of mtDNA content between groups (calculated via Dunn’s post-hoc procedure following Kruskal-Wallis analysis of variance).

	HEU with ASD	HEU without ASD	HUU with ASD
HEU without ASD	**0.011 (0.021)**	-	-
HUU with ASD	**0.002 (<0.001)**	0.38 (0.21)	-
HUU without ASD	**<0.001 (<0.001)**	**0.006 (0.005)**	0.063 **(0.034)**

Numbers in brackets signify *p*-values from Mann-Whitney U-test or Student’s *t*-test. Statistically significant *p* values are bold. -: not applicable.

**Table 4:** Distributions of age, sex, and mtDNA content for three matched groups of HEUs without ASD used in a sensitivity analysis of possible relationship between mtDNA content and ethnicity.

	Asian/South Asian *n* = 10	White/Caucasian *n* = 20	Black/African-Canadian *n* = 20
Male sex	5 (50)	10 (50)	10 (50)
Age at blood collection (years)	1.3 (0.6–2.5) [0.3–15.1]	1.4 (0.5–2.6) [0.2–15.7]	1.3 (0.5–2.6) [0.3–15.6]
MtDNA content	128 (115–196) [90–203]	160 (99–191) [54–259]	169 (133–192) [62–268]

Data reported as median (interquartile range) [range] or *n* (%).

## Supplementary Materials

The following are available online at http://www.mdpi.com/1999-4915/10/2/77/s1, Table S1: Forward and reverse primer sequences used to measure mtDNA content via monochrome multiplex qPCR, Table S2: Thermal cycler settings for monochrome, multiplex qPCR of nuclear (albumin) and mitochondrial (D-loop) sequences.
