# Supplementary material for: Blood Mitochondrial DNA Content in HIV-Exposed Uninfected Children with Autism Spectrum Disorder
Source: Viruses. 2018 Feb 11;10(2):77. doi: 10.3390/v10020077 (PMC5850384; doi:10.3390/v10020077)
Supplement: Supplementary file 1 [file viruses-10-00077-s001.zip › Table S2.docx]

Table S2. Thermal cycler settings for monochrome, multiplex qPCR of nuclear (albumin) and mitochondrial (D-loop) sequences.

| Program | No. of cycles | Target temp (°C) | Acquisition mode | Hold time (mm:ss) | Temp. ramp rate (°C/s) |
| --- | --- | --- | --- | --- | --- |
| Pre-incubation | 1 | 95 | None | 15:00 | 4.4 |
| Amplification | 40 | 94 | None | 00:15 | 2.2 |
|  |  | 62 | None | 00:10 | 2.2 |
|  |  | 74 | Single | 00:15 | 4.4 |
|  |  | 84 | None | 00:10 | 4.4 |
|  |  | 88 | Single | 00:15 | 4.4 |
| Melting Curves | 1 | 95 | None | 01:00 | 4.4 |
|  |  | 45 | None | 00:01 | 2.0 |
|  |  | 95 | Continuous | --- | --- |
| Cooling | 1 | 40 | None | 00:01 | 1.5 |
