# Supplementary material for: Blood Mitochondrial DNA Content in HIV-Exposed Uninfected Children with Autism Spectrum Disorder
Source: Viruses. 2018 Feb 11;10(2):77. doi: 10.3390/v10020077 (PMC5850384; doi:10.3390/v10020077)
Supplement: Supplementary file 1 [file viruses-10-00077-s001.zip › Table S1.docx]

Table S1. Forward and reverse primer sequences used to measure mtDNA content via monochrome multiplex qPCR.

| Gene | Sequence |
| --- | --- |
| Albumin | 5’-CGGCGGCGGGCGGCGCGGGCTGGGCGGAAATGCTGCACAGAATCCTTG-3’ |
|  | 5’-GCCCGGCCCGCCGCGCCCGTCCCGCCGGAAAAGCATGGTCGCCTGTT-3’ |
| D-loop | 5’-ACGCTCGACACACAGCACTTAAACACATCTCTGC-3’ |
|  | 5’-GCTCAGGTCATACAGTATGGGAGTGRGAGGGRAAAA-3’ |

Primers were purified via high-performance liquid chromatography (Integrated DNA Technologies)
